# Decreased DUSP26 Expression Promotes Malignant Behavior in Glioblastoma Cells *via* Deregulation of MAPK and Akt Signaling Pathway

**DOI:** 10.3389/fonc.2021.622826

**Published:** 2021-02-25

**Authors:** Jiajia Chen, Yuecan Zeng, Rong Wu, Ying Xuan, Min Jiang, Hao Teng

**Affiliations:** ^1^ Department of Oncology, Shengjing Hospital of China Medical University, Shenyang, China; ^2^ Department of Neurosurgery, Shengjing Hospital of China Medical University, Shenyang, China

**Keywords:** apoptosis, senescence, proliferation, YAP, glioblastoma

## Abstract

**Purpose:**

Dual-specificity protein phosphatases 26 (DUSP26) is a recently identified phosphatase enzyme that regulates MAPK and Akt signaling pathways. The role of DUSP26 in the development and prognosis of high-grade gliomas (HGGs) and primary glioblastoma (GBM) has remained unclear and was the focus of this study.

**Materials and Methods:**

The prognostic value of DUSP26 was assessed using retrospective analyses using online data sets and tissue microarray of HGGs. U251 and U87 cells modified to overexpress DUSP26 were utilized to study the role of DUSP26 in cell growth, migration, and cell apoptosis analyzed by CCK-8 assay, clonogenic, transwell migration, and TUNEL, respectively. The phosphorylation of proteins in MAPK and Akt signaling pathways was assayed by Western blot and immunofluorescence assays.

**Results:**

Analyses using available online data sets and tissue microarray showed that DUSP26 is down-regulated in high-grade gliomas and GBM as compared to normal brain. Stratification of glioma patients based on DUSP26 expression level showed an inverse correlation between DUSP26 expression and patient survival. At the cellular level, DUSP26 overexpression led to decreased cell proliferation, migration, and senescence in U251 and U87 cells, whereas apoptosis was increased as compared to corresponding controls. Interestingly, the biologic effects of DUSP26 overexpression were associated with the dephosphorylation of proteins in the MAPK and Akt signaling pathways.

**Conclusions:**

These findings suggest that the loss of DUSP26 expression, seen in a subset of high-grade gliomas and GBM patients, facilitates malignant behavior; and with inverse correlation between its expression levels with patient survival. DUSP26 can serve as an independent prognostic factor.

## Highlights

1. This is the first study that verifies the tumor suppressor role of DUSP26 in GBM.
2. High expression DUSP26 correlates with low histology stage and better survival in human HGGs.
3. Overexpression of DUSP26 inhibits malignant behavior *via* dephosphorylation of MAPK and Akt signaling pathway.

## Introduction

High-grade gliomas (HGGs), especially the glioblastomas (GBM), represent the most aggressive primary brain cancer in adults. Although extensive efforts have been devoted to develop new treatments, the mortality from HGGs remains high (1). Recently, high throughput genomic and proteomic analyses of GBM tumors have revealed a number of potential drug targets, including receptor tyrosine kinase (RTK)/RAS/PI3K signaling, p53 signaling, and Rb signaling. However, monotherapies against signaling pathways have proven ineffective, except for some random cases of favorable outcomes (2). While intratumoral heterogeneity and the existence of multiple compensatory signaling have been shown to undermine the efficacy of targeted therapies, molecular mechanisms that trigger and/or propagate compensatory signaling pathways in GBM are not well understood.

Activation of the mitogen‐activated protein kinase (MAPK) signaling pathway, which includes c‐Jun NH2‐terminal kinase (JNK), the p38 MAPK, and the extra‐cellular signal‐related kinase (ERK), has been implicated in the development and progression of HGGs, including GBM (3). Moreover, the large-scale genomic analyses have uncovered activating mutations in the PI3K-Akt signaling pathway (4). Although a number of inhibitors targeting MAPK and AKT signaling pathways have been developed, and despite promising results in preclinical studies or these agents had limited efficacy in clinical trials in patients with GBM (5, 6). MAPK and Akt signaling play a pivotal role in regulating tumor cell proliferation, migration, apoptosis, as well as cell senescence (7). Although MAPK and Akt are independently activated, signaling events often converge in a spatiotemporal manner, and crosstalk between signaling pathways is known to facilitate tumor growth and progression (7). Understanding molecular mechanisms that allow sustained pro-growth signaling in cancer cells is imperative to facilitate drug development.

Dual-specificity protein phosphatases (DUSPs) are protein-tyrosine phosphatases (PTPs) that catalyze the dephosphorylation of protein phospho-serine/threonine and phospho-tyrosine residues. DUSP26 is a recently identified DUSP protein, and its functions are still unclear (8). It has been demonstrated that the mRNA levels of DUSP26 are downregulated in primary GBM as compared to those in the normal brain (8, 9). Since activation of MAPK and Akt signaling pathways depends on a cascade of synchronized phosphorylation and dephosphorylation events, we hypothesized that loss of DUSP26 might contribute toward sustained activation of the MAPK and Akt signaling pathways in HGG cells, where the loss of DUSP26 expression may promote malignant behavior. To address this hypothesis, we have analyzed the biologic effects of DUSP26 overexpression in GBM cells and have retrospectively analyzed the prognostic role of DUSP26 using a tissue microarray developed from glioma patients with known histology and characteristics. Ultimately, we have characterized tumor suppressor role of DUSP26 and highlights potential application of DUSP26 expression profiling as a prognostic marker in glioma patients.

## Materials and Methods

### Bioinformatics Analysis of DUSP26 in GBM

The Gene Expression Profiling Interactive Analysis (GEPIA) is a recently established web-based tool for gene expression analysis between normal tissues and tumor tissues (10). We used GEPIA to analyze DUSP26 expression in GBM *versus* normal brain tissue. To examine heterogeneity in DUSP26 expression, we used an open-source single-cell RNA sequence database (http://gbmseq.org/search) (11). The single-cell RNA sequence database comprises gene expression data for individual cells from tumor core and periphery from four patients with confirmed cases of primary GBM. Cells were re-clustered into neoplastic cells, oligodendrocytes, neurons, astrocytes, vascular cells, myeloid cells, and oligodendrocyte precursor cells (OPCs) by using specific cell markers (11). We checked the distribution of DUSP26 expression in various cell types in human GBM tumors.

### Tissue Microarray of Human Glioma

Human glioma TMA was obtained from National Engineering Center for Biochip at Shanghai (Shanghai, China, #HBraG180Su02). The TMA contains 180 cases of the glioma tissue collected from patients who underwent surgical resection of glioma between February 2008 and November 2011. The clinical parameters were collected, and patients were followed up after surgery till February 2018. Written informed consent was obtained from all patients before participation in the study. All the tissue samples were fixed with formalin, embedded in paraffin, and sectioned by the manufacturer. The study was approved by the ethical committee of Shengjing Hospital, China Medical University.

### Immunohistochemistry

TMA sections were deparaffinized in xylene and rehydrated in a series of ethanol dilutions. Antigen retrieval was performed by heating sections with 10 mM sodium citrate buffer. Subsequently, sections were blocked with 10% goat serum and then incubated with primary DUSP26 antibody (1:100, Abcam #ab224407) overnight at 4°C. Next, sections were incubated with secondary antibody for 30 min, and finally stained with 3,3′-diaminobenzidine tetrahydrochloride solution, and counter-stained with hematoxylin.

### IHC Scoring

Two independent pathologists blinded to clinical and pathologic information assessed the expression of DUSP26 on the TMA slide. The expression of DUSP26 in glioma tissues was scored semi-quantitatively combining positive percentage and intensity of stained sections (staining index=positive×intensity score) according to previous literature (12, 13). The positivity was scored as 0 if no detectable staining was observed; 1 if positivity was <20%; 2 if positivity was 20–75% and 3 if positivity>75% was observed. The intensity of stained tumor cells was graded on the following scale: 0, negative; 1, weak; 2, moderate; 3, strong staining. Based on the staining index, a final total score of the top 50% cases was considered high DUSP26 group, whereas others were defined as the low DUSP26 group.

### Cell Culture and Treatment

Human malignant glioma U251 and U87 cells were obtained from the Chinese Academy of Medical Sciences (Beijing, China). Cells were cultured in DMEM/F12 medium supplemented with 10% fetal bovine serum (FBS, Gibco #26140079) and 1% penicillin/streptomycin. Cells were incubated at 37°C in a humidified incubator with an atmosphere of 5% CO2.

### DUSP26 Overexpression Plasmid Transfection

U251 and U87 cells were seeded in six-well plates with a density of 70% and cultured overnight. DUSP26 overexpression plasmid (Origene #RC200202) corresponding empty vector were transfected into U251 and U87 cells by using Lipo3000 reagent (Thermo Fisher #L3000015), as per instruction manual. Briefly, 5µg plasmids were diluted in 250 µl OptiMEM and then mixed with 2.5 µl of P3000 reagent. Then, the mixture was added to cultured U251 and U87 cells. The cells were harvested after 48 h, the mRNA and protein levels of DUSP26 were analyzed using quantitative PCR (qPCR) and Western blot.

### Quantitative PCR

Total RNA was extracted using the RNeasy Mini Kit (Qiagen #74104) according to the manufacturer’s protocol. An equal amount of total RNA (500 ng) was reverse-transcribed to cDNA using the SuperScript III kit (Invitrogen #18080-051). Then, qPCR was performed using the SYBR Green Supermix (Bio-Rad #1725120). The primers of human DUSP26 (NM_001305115.2), p21 (NM_000389.5), p16 (NM_000077.5), and GAPDH (NM_001256799.3) were synthesized by Invitrogen. GAPDH forward: 5′-ACATCGCTCAGACACCATG-3′, reverse: 5′-TGTAGTTGAGGTCAATGAAGGG-3′; DUSP26: forward: 5′-ATCTCGGAGACCAGGACAT-3′, reverse: 5′-CTCAACACCCAGGTAGCG-3′.

P21 forward: 5′-TGTCACTGTCTTGTACCCTTG-3′; reverse: 5′- GGCGTTTGGAGTGGTAGAA-3′. P16 forward: 5′-GATGTCGCACGGTACCTG-3′; reverse: 5′-TCTCTGGTCTTTCAATCGGG-3′. The protocol was performed by using a standard qPCR program, which consisted of 40 cycles of 95°C for 15 s and 60°C for 30 s. Expressions were normalized to GAPDH, and fold-changes were calculated by the relative quantification (2^−ΔΔCt^) method.

### Immunofluorescence

U251 or U87 cells were seeded on coverslips in the six-well plates and incubated overnight. Cells were washed with PBS, fixed in 4% paraformaldehyde for 15 min, permeabilized with 0.5% Triton X-100 and blocked with 10% FBS for 1 h. Then cells were incubated with the primary antibody overnight at 4°C as listed in **Supplementary Table 1** ****. After washing with PBS 3 times, cells were incubated with a secondary antibody conjugated to Alexa Fluor 488 or Alexa Fluor 546 for 1 h at room temperature in the dark. Finally, coverslips were mounted onto the glass slides using an antifade reagent containing DAPI to counterstain nuclei. Fluorescently labeled cells were observed under a confocal microscope (Zeiss #LSM510) using 400X objective lens. Images were captured using a CCD camera attached to the microscope.

### Transwell for Migration

Cell migration ability was evaluated by a 24-well transwell chamber with 8 µm pore size (Corning #CLS3464). Cells were suspended in 100 μl serum-free medium and added to the upper chamber, and cultured for 4 h. The lower chamber was then filled with 500 μl medium containing 10% FBS, and the upper chamber was inserted into a lower chamber, 8 h after incubation. Non-migrated cells were wiped from the upper surface of the transwell membrane, and cells that migrated to the underside of the membrane were fixed with methanol and stained with 20% Giemsa and counted under a microscope.

### Wounding Healing Assay

Cells (1x10^6^) were seeded in six‐well plates and cultured overnight to form a monolayer. The monolayer was gently scratched with a 200 μl sterile pipette tip to create a wound field. After washing with PBS, cells were cultured in fresh medium for 24 h. Images of the scratches were captured with the microscope at 0- and 24-h time points, and the extent of wound closure was determined.

### Cell Proliferation Assay

The cell proliferation ability was measured by the CCK-8 assay. Cells were seeded into 96-well plates at a density of 2,000 cells per well. After 72 h, CCK-8 (Dojindo, #CK04) reagent was added (10 μl/well) and then incubated for 1–2 h. The proliferation was quantified by measuring absorbance at 450 nm in a SpectraMax M5 microplate reader (Molecular Devices, San Jose, USA).

### Clonogenic Assay

U251 cells (500 cells per well) were seeded in six-well plates for clonogenic assay. Cells were cultured at 37°C in a humidified incubator with a 5% CO_2_ atmosphere for 14 days. Colonies were fixed and then stained with crystal violet. Colonies comprised of >50 cells, were manually counted, photographed using a handheld camera.

### Terminal Deoxynucleotidyl Transferase dUTP Nick End Labeling

Apoptosis was analyzed by using the TUNEL Assay Kit (Roche Diagnostics #11684795910). Cells fixed in 4% paraformaldehyde for 15 min at room temperature were permeabilized with 0.5% Triton X-100 and subsequently labeled with a TUNEL reaction mixture as per the instruction manual for 1 h at 37°C in a humidified atmosphere in the dark. After three wash with PBS, cells were counter-stained by DAPI. Images of five random fields under a microscope with × 400 magnifications were captured. The TUNEL-positive nuclei were counted, and percentages of TUNEL-positive cells were calculated.

### Western Blot

Whole-cell pellets were homogenized in a RIPA lysis buffer (Cell signaling #9806S) with a protease inhibitor cocktail (Roche #4693159001) and phosphatase inhibitor cocktail (Sigma #P0044). An equal amount (10 to 15 μg) of proteins were separated in 4–12% SDS-PAGE gel and then transferred onto the PVDF membrane. The membranes were blocked by 5% non-fat dry milk for 1 h and then incubated overnight at 4°C with appropriate primary antibodies (listed in **Supplementary Table 1**). Next, the membranes were incubated with horseradish peroxidase-conjugated secondary antibodies for 1 h at room temperature. Immunoblots were visualized by an enhanced chemiluminescence kit (Santa Cruz #sc-2048) and autoradiography. HSC70 was regarded as an internal control to calculate relative integrated density values.

### Statistical Analysis

Quantitative data were expressed as mean ± standard deviation. The student *t*-test was used for statistical comparisons. The Kaplan-Meier method was utilized to depict patient survival, which was compared by the log-rank test. The χ^2^ test was applied to analyze categorical variables. All data were analyzed by SPSS 19.0 software (SPSS, Chicago, IL, USA). A *p*-value of < 0.05 was considered significant.

## Results

### Bioinformatics Analysis Indicates Down-Regulation of DUSP26 in GBM

**Figure 1:**
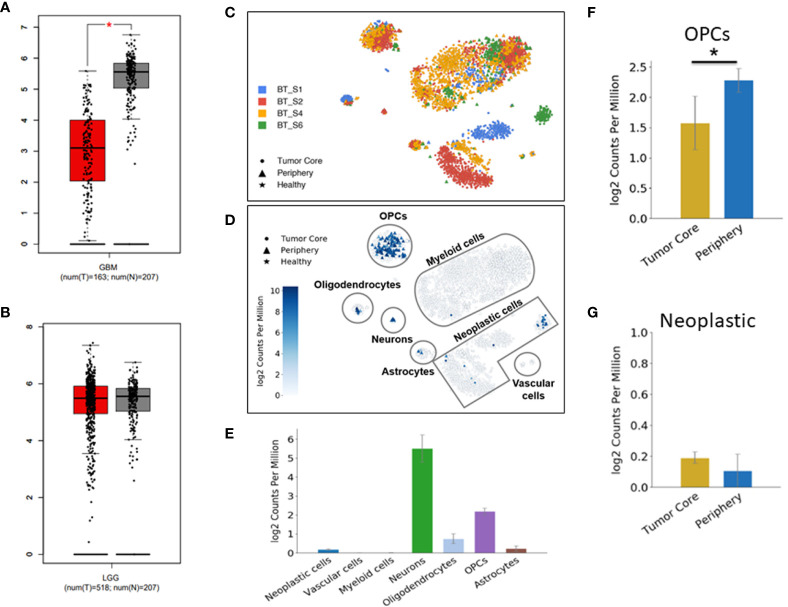
Down-regulation of DUSP26 in glioblastoma (GBM). **(A, B)** Box-whisker plots showing mRNA expression of DUSP26 in GBM *versus* normal brain, *p<0.01 **(A)** and that in low-grade gliomas *versus* normal brain tissues **(B)** as per GEPIA database. **(C)** Cluster of cells as defined by cell-type-specific markers in a single-cell RNA sequencing data from four-GBM patients. Each color represents the cell population from an individual patient. **(D)** Dot plots showing DUSP26 expression in different cell types and locations within a GBM tissue, as analyzed by single-cell RNA sequencing. **(E)** The graphs showing the distribution of DUSP26 transcript in various cell types; ● indicate tumor core cells, ▲ indicate periphery cells, ★indicate healthy cells, the gradient colored scale bar represents relative DUSP26 expression levels. **(F)** Graphs showing relative DUSP26 mRNA levels in oligodendrocyte precursor cell (OPC) cells obtained from the tumor core and periphery, *p < 0.05. **(G)** Graphs showing DUSP26 mRNA levels in neoplastic cells from the tumor core and periphery.

To assess DUSP26 expression levels in GBM *versus* normal brain tissue, we used the GEPIA database, which allows tumor/normal differential expression analysis (10). As shown in **Figure 1A**, the mRNA level of DUSP26 is significantly decreased in the GBM tumor biopsies (N=163) as compared to that in normal brain tissues (N=207), whereas, the DUSP26 mRNA levels in low-grade gliomas (LGG) tumor biopsies (N=518) did not differ from that in normal brain tissue (**Figures 1A, B**). These results suggest that decreased DUSP26 expression is a characteristic feature of malignant brain tumors.

Since normal cells within tumor microenvironment may also account toward DUSP26 expression profiling by RNA-sequencing, therefore, estimate to DUSP26 mRNA seen in GBM tissues may not be a true estimate of DUSP26 levels in GBM cells. To address this notion, the single-cell RNA sequence data set (available online) was used to determine inter and intra-tumoral heterogeneity of DUSP26 expression in GBM. As shown in **Figure 1C**, each GBM tumor comprised cells types that were clustered based on specific cell markers. Interestingly, DUSP26 was mainly expressed in normal brain cells located within the tumor region such as neurons, oligodendrocyte precursor cell (OPC) cells, and oligodendrocytes, whereas, only a fraction of neoplastic cells from one of four brain tumors had DUSP26 expression, while neoplastic cells from remaining three tumors had negligible DUSP26 expression (**Figure 1D**). Based on quantification, DUSP26 expressed at the highest level in neurons, followed by OPCs and oligodendrocytes. Interestingly, DUSP26 levels in neoplastic cells and astrocytes were modest, while vascular endothelial cells and myeloid cells had no appreciable DUSP26 expression (**Figure 1E**). OPCs, which were throughout the tumor region, had varied DUSP26 expression based on their location within the tumor (**Figure 1F**). However, unlike OPCs, DUSP26 expression in neoplastic cells did not vary based on their location (**Figure 1G**). In summary, DUSP26 expression is down-regulated in neoplastic cells within GBM tissue, where non-tumor cells account for the bulk of DUSP26 expression.

### DUSP26 Expression Correlates With the Progression of Human GBM

As DUSP26 is down-regulated in GBM but not in low-grade gliomas (**Figure 1B**), we next checked whether the expression of DUSP26 correlates with the progression of human gliomas. We used a GBM tissue microarray comprising of tumor tissues from 180 cases of glioma patients, 110 cases were male (71.1%), and 70 cases were female (38.9%). The average age was 41.1 ± 17.3 years old, with 127 cases younger than 50 (70.6%) and 53 cases older than 50 (29.4%). For the tumor’s location, 93 cases (51.7%) were found in the left brain, and 87 cases (48.3%) were found in the right brain. The histology stage of 107 cases was identified as stage I–II (59.4%), and 73 cases were identified as stage III–IV (40.6%).

Based on the expression of DUSP26, 180 cases were divided into the low DUSP26 group and the high DUSP26 group, with 90 cases in each group. There was no significant difference in age, gender, and location between the low *versus* high DUSP26 group (**Table 1**, *p*>0.05). Interestingly, the low DUSP26 group had only 39 cases (43.3%) of low grade (stage I˜II) gliomas, while 51 cases (56.7%) were HGGs (stage III˜IV). Unlike the low DUSP26 group, the high DUSP26 group had 68 cases (75.6%) of low grade (stage I˜II), while only 22 cases (24.4%) were HGG (stage III˜IV), indicating that low DUSP26 expression is associated with advanced histologic stages of gliomagenesis (**Table 1**, *p*<0.0001). In summary, high DUSP26 expression correlates with the low histologic stages of glioma, while DUSP26 is down-regulated or suppressed in HGGs, which include GBM.

**Table 1:** Relationships between DUSP26 expression and clinicopathological characteristics in 180 glioblastoma (GBM) cases.

Characteristics	DUSP26 expression (%)		*p*-value
	Low	High	
Total	90	90	
Average years	43.0 ± 17.9	39.2 ± 16.6	
<50	59 (65.6)	68 (75.6)	0.095
≥50	31 (34.4)	22 (24.4)
Gender			
Male	57 (63.3)	53 (58.9)	0.323
Female	33 (36.7)	37 (41.1)
Histologic grade			
I-II	39(43.3)	68 (75.6)	<0.0001
III-IV	51 (56.7)	22 (24.4)
Location (cm)			
Left brain	42 (46.7)	51 (56.7)	0.116
Right brain	48 (53.3)	39 (48.3)

### High Expression of DUSP26 Associates With Better Survival in Glioma Patients

**Figure 2:**
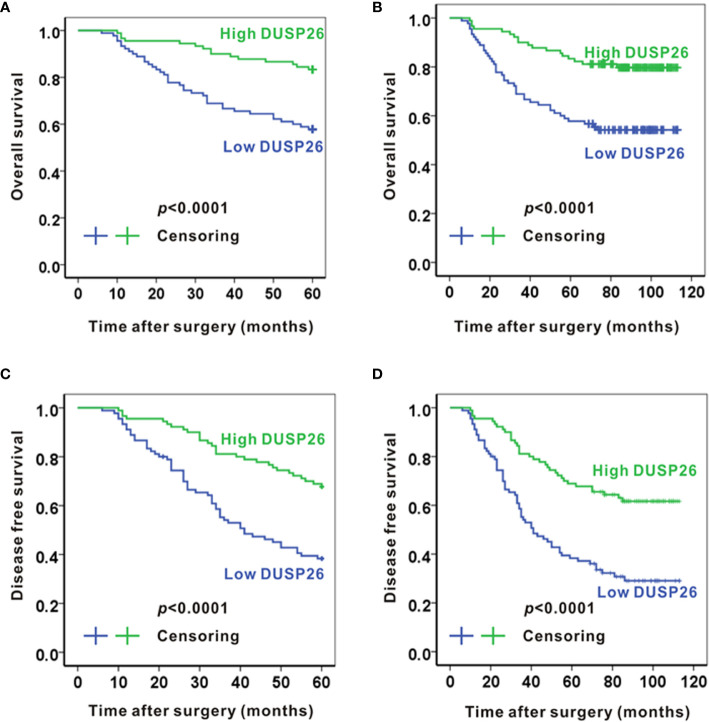
High expression of DUSP26 associates with better survival in glioma patients. **(A, B)** Kaplan-Meier graphs showing overall 5-year survival **(A)** and overall 10-year survival **(B)** for patients stratified based on DUSP26 expression levels by IHC. **(C, D)** Kaplan-Meier graphs showing disease-free 5-year survival **(C)** and disease-free 10-year survival **(D)** for the same patients analyzed in **(A, B)**, the p values are based on log-rank test.

As the advanced histology stage historically predicts poor survival in glioma patients, we asked if there is any relationship between DUSP26 expression and patient survival. Consistent with a mixed population of glioma patients comprising those of grade I–IV, the 5-year overall survival rate was 70.6%. The average 5-year overall survival was 50.9 months. The average 5-year overall survival time for the low and high DUSP26 groups was 46.2 and 55.5 months, respectively. Moreover, the overall 5-year survival rates were 57.8 and 83.3% for the patients with low and high DUSP26 groups, respectively. When stratified by the DUSP26 expression level, patients with high DUSP26 had significantly better overall 5-year survival as compared to those with low DUSP26 expression (*p*<0.0001, **Figure 2A**). The 10-year overall survival rate was 67.2%, with an average overall survival time of 86.7 months. Moreover, the overall 10-year survival rate was 54.4% in the low DUSP26 group as compared to 80.0% in the high DUSP26 group (*p*<0.0001, **Figure 2B**). The disease-free 5-year survival rate was 38.9% in the low DUSP26 group and 67.8% in the high DUSP26 group of patients. Similarly, the disease-free 10-year survival rates were 31.1 and 62.2% in the low and high DUSP26 groups, respectively. Patients with high DUSP26 expression had better disease-free 5- and 10-year survival rates compared that for patients with low DUSP26 expression (*p*<0.0001, **Figures 2C, D**). In summary, the high DUSP26 expression is associated with better survival in glioma patients regardless of their age, sex, and treatment history.

### Exogenously Expressed DUSP26 Inhibits Proliferation and Migration in GBM Cells

**Figure 3:**
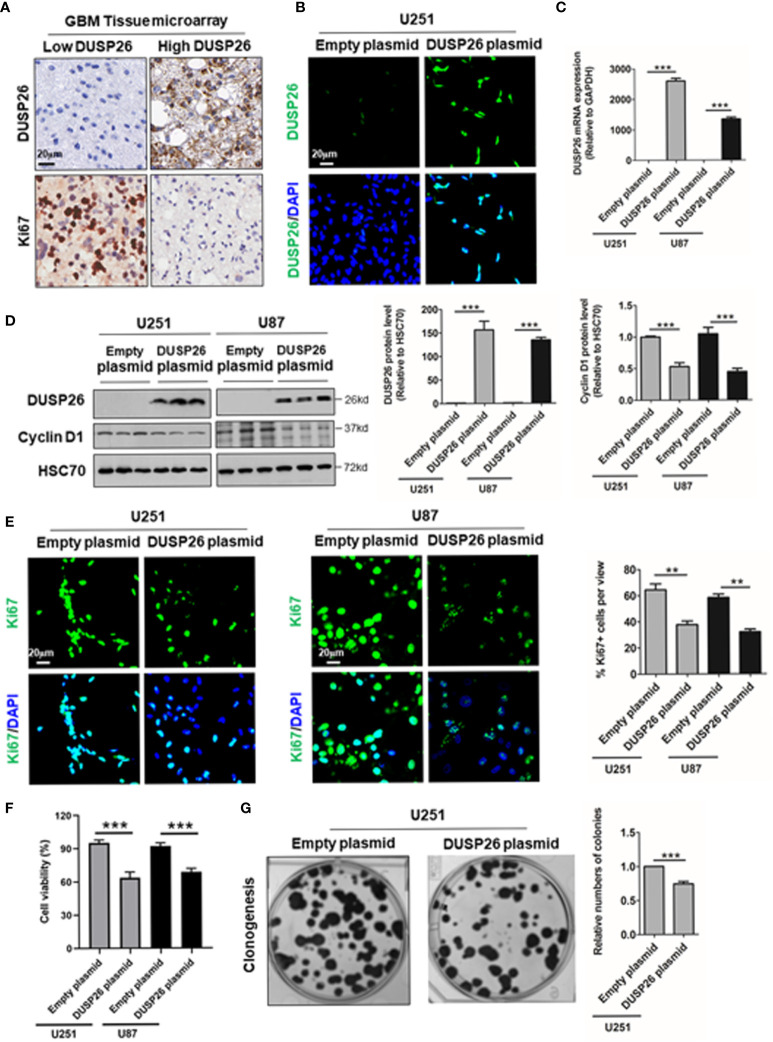
Effect of DUSP26 overexpression on cell proliferation in glioblastoma (GBM) cells. **(A)** Representative images showing DUSP26 and Ki67 expression in tissue microarray slide subjected to IHC. **(B)** Representative fluorescent images showing DUSP26 expression in U251 cells transfected with empty vector or DUSP26 encoding plasmid, cells were analyzed 48 h after transfection, and nuclei (blue) were counterstained with DAPI. **(C)** Bar graphs showing relative levels of DUSP26 mRNA (normalized to GAPDH) in U251 or U87 cells transfected with empty vector or DUSP26 encoding plasmid, cells were analyzed 48 h after transfection by qRT-PCR. **(D)** Representative images of western blots (panels on the left side) showing levels of DUSP26 and cyclin D1 in lysates of U251 or U87 cells transfected with empty vector or DUSP26 plasmid construct, and lysed 48 h after transfection were analyzed in triplicate, HSC70 was used as loading control; graphs on the right show the quantification of DUSP26 levels (middle) and cyclin D1 (right) in western blots in the left panel. **(E)** Representative fluorescent images showing Ki67 in U251 or U87 cells transfected with empty vector or DUSP26 encoding plasmid, cells were analyzed 48 h after transfection, and nuclei (blue) were counterstained with DAPI, shown on the right side are bar graphs of quantification of Ki67 positivity based on the scoring of 300 nuclei per line. **(F)** The cell proliferation of U251 and U87 cells transfected with empty vector or DUSP26 encoding plasmid were quantified by CCK-8 assay. **(G)** Representative images showing clonogenic growth (left) in U251 cells transfected with empty vector or DUSP26 encoding plasmid; and bar graphs on the right present quantification of colony formation; **p < 0.01; ***p < 0.001.

As high DUSP26 expression predicts better survival and lower histology stage in human glioma patients, we next checked the effects of DUSP26 overexpression on proliferation and migration of GBM cells, where basal levels of DUSP26 are low. In the GBM tissue microarray, we found that DUSP26 was mainly expressed in the nuclei of tumor cells (**Figure 3A**), and the expression of DUSP26 correlates with Ki67 staining in GBM (**Supplementary Table 2**). 36.7% and 23.3% of cases showed more than 5% positive cells of Ki67 in patients with low DUSP26 group and high DUSP26 group, respectively (*p*=0.037). However, the DUSP26 expression showed no significant correlation with other gene expressions, such as EGFR, PDL1, GFAP, S100, CD34, EMA, CK, and vimentin (*p*>0.05, **Supplementary Table 2**).

As high DUSP26 expression associates with low histologic stage and better prognosis in glioma patients, we next investigated the anti-tumor effect of DUSP26 *via* overexpression of DUSP26 in the U251 and U87 GBM cells. The DUSP26 overexpression over control cells was confirmed by IF, qPCR, and WB (**Figures 3B–D**). The overexpressed DUSP26 was mainly located in nuclei (**Figure 3B**), which is consistent with the IHC based detection of DUSP26 in GBM patients (**Figure 3A**). As a proliferation maker, cyclin D1 was significantly inhibited after DUSP26 overexpression in U251 and U87 cells (**Figure 3D**). Also, the percentage of Ki67 positive cells was significantly decreased after DUSP26 overexpression in U251 and U87 cells (**Figure 3E**). Furthermore, the cell proliferation quantified by CCK-8 assay was also significantly decreased after DUSP26 overexpression in U251 and U87 cells as compared to corresponding controls (**Figure 3F**). Similarly, clonogenic potential in U251 cells was significantly after DUSP26 overexpression decreased in U251 cells as compared to control cells (**Figure 3G**). These results indicate that DUSP26 expression in glioma cells may control malignant behavior by decreasing cell proliferation.

**Figure 4:**
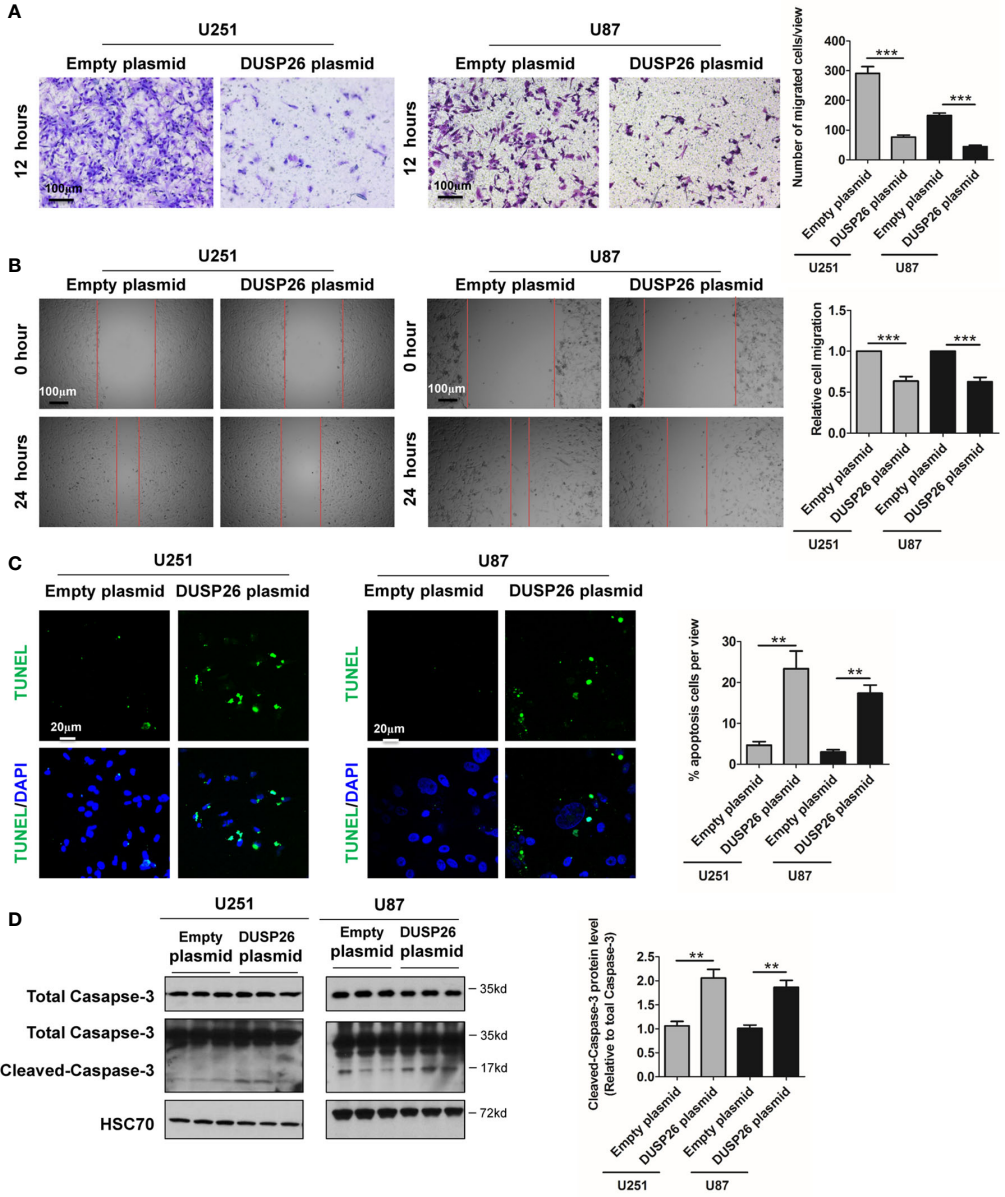
Effect of DUSP26 overexpression on cell migration and apoptosis in glioblastoma (GBM) cells. **(A)** Representative images (left side) and graphs (right side) showing relative cell migration as determined by transwell migration assays using U251 and U87 cells transfected with an empty plasmid or DUSP26 expression plasmid. Cells were allowed to migrate for 8 h. Cells migrated cells were stained and imaged under a microscope using 10X objective lenses and quantified. **(B)** Photomicrographs showing relative wound healing at 0 and 24 h after the wound was scratched in monolayers of U251 and U87 cells transfected with an empty plasmid or DUSP26 expression plasmid wound closure was measured and quantified. **(C)** Representative images (panels on the left side) and graphs of quantification (panel on the right side) of apoptotic cells detected by TUNEL assay performed using U251 and U87 cells transfected with an empty plasmid or DUSP26 expression plasmid. **(D)** Representative images of western blots (panels on the left side) and showing levels of total caspase-3, cleaved caspase-3 in lysates of U251 or U87 cells transfected with empty vector or DUSP26 plasmid construct, and lysed 48 h after transfection were analyzed in triplicate, HSC70 was used as loading control; graphs depicted on the right side show the quantification of cleaved caspase-3. **p < 0.05; ***p < 0.001.

In addition to proliferative potential, migration capacity in cancer cells also reflects malignant behavior, and both MAPK and Akt signaling pathways play crucial roles in orchestrating cell migration (5–7). To understand the role of DUSP26 in the prevention of malignant behavior, we tested the effect of DUSP26 on cell migration. In a transwell migration assay, the number of cells migrated through the transwell membrane, was significantly reduced after overexpression of DUSP26 in U251 and U87 cells, as compared to control cells (**Figure 4A**). Wound healing assays, where migratory cells can rapidly fill the gap areas, were used as an alternative measure of cell migration. In a wound healing assay also the migration rate was markedly decreased after DUSP26 overexpression in U251 and U87 cells as compared to control cells (**Figure 4B**). In summary, overexpression of DUSP26 in GBM cells can undermine malignant behavior by inhibiting cell proliferation and migration capacity.

### Overexpression of DUSP26 Increase Apoptosis and Reduces Cell Senescence

**Figure 5:**
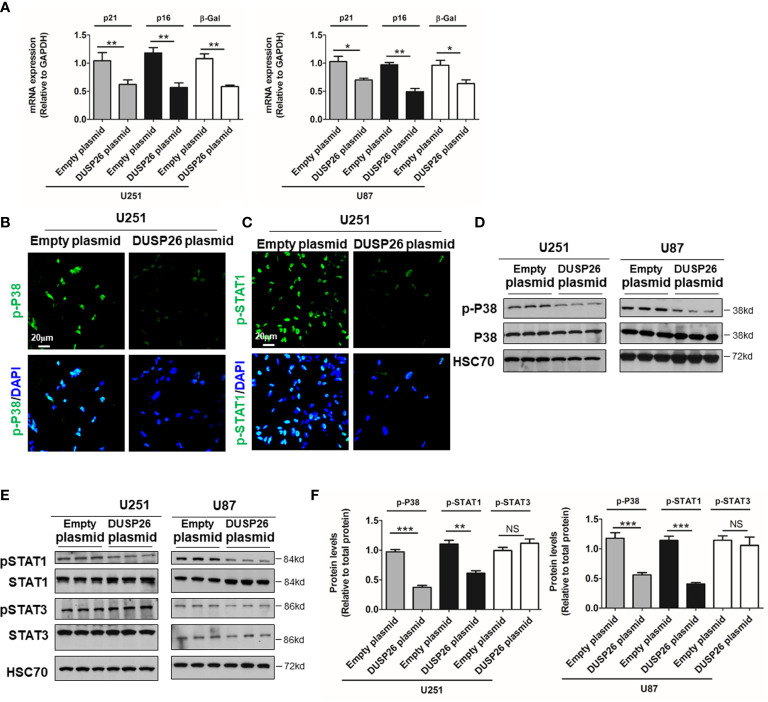
Effect of DUSP26 overexpression on the preponderance of senescence in glioblastoma (GBM) cells. **(A)** Graphs showing relative expression of classical markers senescence-p21, p16, and β-Gal in U251 and U87 cells transfected with empty plasmid or DUSP26 expression construct. **(B, C)** Representative images of immunofluorescence based detection of p-p38 **(B)** and p-STAT1 **(C)** in U251 cells transfected with an empty vector or DUSP26 expression plasmid, cells were analyzed 48 h after transfection, and nuclei (blue) were counterstained with DAPI. **(D, E)** Representative images of western blots showing levels of p38 and p-p38 (Thr180/Tyr182) **(D)**; STAT1, p-STAT1 (Ser727), STAT3, and p-STAT3 (Tyr705) **(E)**, in lysates of U251 or U87 cells transfected with empty vector or DUSP26 plasmid construct, cells were lysed 48 h after transfection and analyzed in triplicate, HSC70 was used as a loading control. **(F)** Quantification of the Western blots depicted in **(D)** and **(E)**. *p < 0.05; **p < 0.01; ***p < 0.001, NS, non-significant.

Cell apoptosis and cell senescence play a pivotal role in the development of cancer and response to therapy. We next explored the role of DUSP26 in cell apoptosis and cell senescence. In a TUNEL assay, the apoptosis increased from 5 to 36% and 4 to 17% after DUSP26 overexpression in U251 and U87 cells, respectively (*p*<0.001, **Figure 4C**) as compared to corresponding controls. Moreover, cleaved caspase-3, which is also a marker for cell apoptosis, was significantly increased after DUSP26 overexpression in U251 and U87 cells, respectively (*p*<0.001, **Figure 4D**). These results indicate that DUSP26 overexpression can promote cell apoptosis in U251 and U87 cells. The mRNA levels of classical markers of cell senescence, p21, p16, and β-Gal, were significantly reduced after DUSP26 overexpression in U251 and U87 cells (**Figure 5A**). Moreover, as p38, STAT1, and STAT3 are involved in cell senescence, the phosphorylated p38 (p-p38) and phosphorylated STAT1 (p-STAT1) were markedly decreased, whereas the phosphorylated STAT3 (p-STAT3) remained unchanged after DUSP26 overexpression in U251 and U87 cells (**Figures 5B–F**). In summary, the overexpression of DUSP26 in GBM cells can promote cell apoptosis and by preventing cells from senescence.

### DUSP26 Regulates Dephosphorylation of Proteins in MAPK and Akt Signaling Pathway

**Figure 6:**
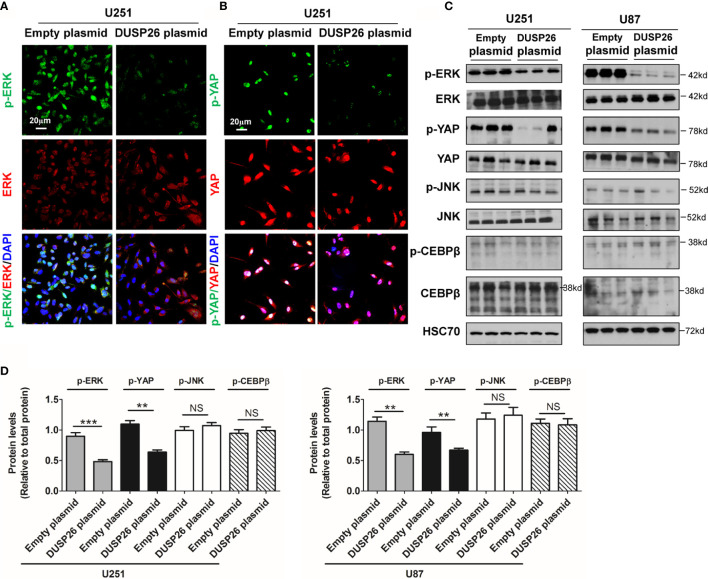
Effect of DUSP26 overexpression on the MAPK signaling pathway. **(A)** Representative images of immunofluorescence based detection of p-ERK (green), ERK (red) in U251 cells transfected with empty vector or DUSP26 expression plasmid, cells were analyzed 48 h after transfection, and nuclei (blue) were counterstained with DAPI. **(B)** Representative images of immunofluorescence based detection of p-YAP (green), YAP (red) in U251 cells transfected with empty vector or DUSP26 expression plasmid and processed as described under **(A)**. **(C)** Representative images of western blots showing levels of p-ERK (Thr202/Tyr204), ERK, p-YAP (Ser127), YAP, p-JNK (Thr183/Tyr185), JNK, p-CEBPβ (Thr235), and CEBPβ in lysates of U251 or U87 cells transfected with empty vector or DUSP26 plasmid construct, cells were lysed 48 h after transfection and analyzed in triplicate, HSC70 was used as loading control. **(D)** Quantification of the western blots depicted in **(C)**. **p < 0.01; ***p < 0.001 and NS, denotes non-significant.

Many oncogenic signaling pathways, such as the MAPK and Akt signaling pathway, are deregulated/over-activated in GBM. As a member of family of phosphatase enzymes, DUSP26 is known to dephosphorylate a number of signaling proteins. We next checked the effect of DUSP26 on phosphorylated proteins involved in the MAPK and Akt signaling pathways. In the MAPK signaling pathway, the levels of phosphorylated ERK (p-ERK) and phosphorylated YAP (p-YAP), as demonstrated both by IF and WB, were significantly decreased in U251 and U87 cells transfected with DUSP26 plasmid, as compared to those transfected with an empty vector (**Figures 6A–D**), whereas total levels of ERK, YAP, JNK, and CEBPβ did not change (**Figures 6A–D**). Also, the levels of phosphorylated JNK (p-JNK) and phosphorylated CEBPβ (p-CEBPβ) had no differences between cells expressing an empty plasmid *versus* those expressing DUSP26 (**Figures 6A–D**). These results suggest that DUSP26 dephosphorylates only select proteins among the cascade of proteins in the MAPK signaling pathway.

**Figure 7:**
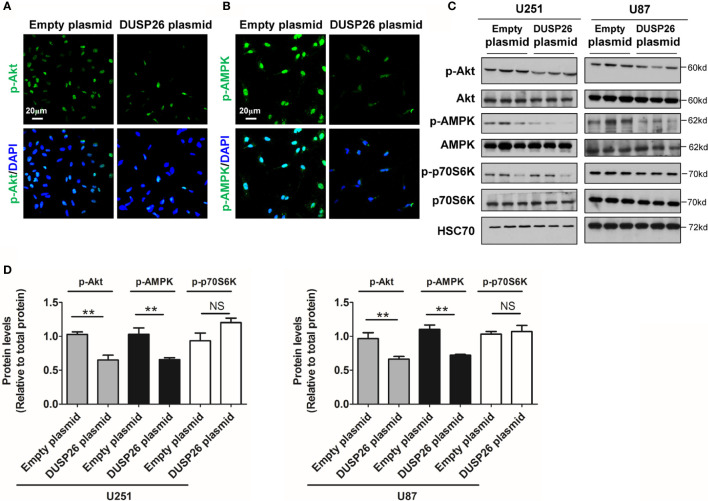
Effect of DUSP26 overexpression on the Akt signaling pathway. **(A)** Representative images of immunofluorescence based detection of p-Akt (green) in U251 cells transfected with empty vector or DUSP26 cassette, cells were analyzed 48 h after transfection, nuclei were counterstained with DAPI. **(B)** Representative images of immunofluorescence based detection of p-AMPK in U251 cells transfected with empty vector or DUSP26 cassette and processed as described under **(A)**. **(C)** Representative images of western blots showing relative p-Akt (Thr308), Akt, p-AMPK (Thr172), AMPK, p-p70S6K (Ser371), and p70S6K in lysates of U251 or U87 cells transfected with empty vector or DUSP26 expression plasmid, cells were lysed 48 h after transfection and analyzed in triplicate, HSC70 was used as a loading control. **(D)** Quantification of the Western blots depicted in **(C)**, **p < 0.01, NS, not significant.

Next, we examined the effect of DUSP26 on the Akt signaling pathway. In the Akt signaling pathway, total levels of Akt, AMPK, and p70S6K were comparable in cells expressing the empty vector *versus* those expressing DUSP26 (**Figures 7A–D**). However, the levels of phosphorylated Akt (p-Akt) and phosphorylated AMPK (p-AMPK) were markedly suppressed by DUSP26 overexpression, as demonstrated by both IF and WB (**Figures 7A–D**). Interestingly, there were no significant difference between cells expressing empty vector *versus* DUSP26 in terms of phosphorylated p70S6K (p-p70S6K) (**Figures 7A–D**). In summary, these results indicate that DUSP26 facilitates dephosphorylation of ERK, YAP, Akt, and AMPK.

## Discussion

GBM is the most aggressive and lethal brain tumor. To improve the clinical prognosis of GBM, it is crucial to explore the cellular and molecular mechanisms of GBM tumors. To our knowledge, this is the first study that verifies the tumor suppressor role of DUSP26 in HGG. In the current study, we addressed the following novel observations: 1) we have identified that the expression of DUSP26 is decreased in GBM tumors, and the high expression DUSP26 correlated with low histology stage, and better survival in glioma patients; 2) overexpression of DUSP26 in U251 or U87 undermines malignant behavior by decreasing cell proliferation, cell migration, and cell senescence, and by promoting cell apoptosis; 3) the anti-tumor effect of DUSP26 overexpression attributed to the inhibition of MAPK and Akt signaling pathway *via* dephosphorylation of p38, STAT1, ERK, YAP, Akt, and AMPK. These findings indicate that DUSP26 can be an excellent prognostic marker, while strategies to restore DUSP26 overexpression in GBM cells can be useful to design novel therapeutic approaches.

After stress or mitogenic stimulation, DUSPs are transcriptionally expressed to inactivate phosphorylated proteins (3). As the potential ability to dephosphorylate proteins, several DUSPs have been shown to play critical roles in human cancers, such as DUSP2 in colon cancer (14), DUSP6 in lung cancer (15), and DUSP8 in hepatocellular carcinoma (16). DUPS26 is a recently identified DUSP that is mainly expressed in the brain. High expression of DUSP26 was detected in the neuropil and neurons in the normal brain, while expression was usually absent or low in GBM tumor tissues (9). Moreover, the mRNA level of DUSP26 was downregulated in human glioblastoma samples (8). Consistently, by using bioinformatics analyses, we found that the mRNA level of DUSP26 was decreased in GBM tissue as compared to that in the normal brain (**Figure 1A**). These results are also confirmed by a single-cell RNA-sequence database, where DUSP26 mRNA was barely detectable in neoplastic cells of one of four GBM samples (**Figure 1B**). Thus, it is predictable that DUSP26 might play a pivotal role in the development or progression of GMB; however, till date, the precise role of DUSP26 in GBM has remained unknown. By correlative analysis of DUSP26 expression with patient survival, we have verified that DUSP26 expression can be predictive of histology stage and survival outcome in human glioma patients. Furthermore, observations that DUSP26 overexpression inhibits tumor cell proliferation and migration, induce cell apoptosis, collectively highlight tumor suppressive role of DUSP26 in HGG. Whether this is true in other malignancies has yet to be seen. Nevertheless, our observations are in line with prior reports showing the anti-proliferative role of DUSP26 in HeLa cells (17).

Malignant cells invading the normal brain tissues is a well-known hallmark of GBM. It is generally accepted that over activation of the survival signaling pathways, such as MAPK and Akt signaling pathways, play a pivotal role in promoting malignant behavior be in GBM cells (18). Whereas the role of DUSP26 has not been studied. In the current study, we found that high expression of DUSP26 has a direct impact on multiple signaling pathways, including MAP kinase and Akt pathways, suggesting that loss of DUSP26 expression is a crucial regulatory event associated with over activation of pro-tumor signaling in GBM cells. JNK, ERK, and p38 are three major MAPK signaling with diverse biological functions. Activation of JNK and ERK are involved in the regulation of GBM proliferation and migration (19). As p-ERK (but not p-JNK) is increased after DUSP26 overexpression in U251 and U87 cells, it is presumable that overexpression of DUSP26 may inhibit cell proliferation *via* suppressing p-ERK. YAP is a highly related transcriptional factor pervasively activated in human GBM (20, 21). Activation of YAP promotes cell proliferation and migration that eventually induces malignant behavior in GBM (21). In this study, DUSP26 overexpression led to dephosphorylation of YAP that presumably inhibited YAP activity. Thus, it is presumable that the anti-tumor effect of DUSP26 overexpression may arise from inhibition of MAPK-ERK and YAP signaling pathway *via* dephosphorylation of ERK and YAP. However, how DUSP26 regulates cell proliferation and migration *via* dephosphorylation of ERK and YAP needs further studies.

The pathways that control cell survival, such as PI3K-Akt, are altered in GBM cells, leading to resistance toward apoptotic stimuli (22). It has been demonstrated that PI3K/Akt signaling is deregulated in nearly 80% of all GBM (23). Phosphorylation of Akt, which activates cascade of downstream targets like AMPK and mTOR eventually induces resistance to apoptotic stimuli (23). In the current study, DUSP26 overexpression decreased phosphorylation Akt and AMPK. However, the phosphorylation of p70S6K, a downstream target of mTOR, did not change after DUSP26 overexpression. Thus, suggesting that down-regulation of Akt-AMPK axis rather than Akt-mTOR, may be involved in the induction of cell apoptosis after DUSP26 overexpression. In accordance with our results, AMPK is highly expressed in GBM and promotes glioblastoma bioenergetics and tumor growth, while inhibition of AMPK suppresses tumor growth by induces GBM cell apoptosis (24, 25). Cellular senescence leads to loss of mitotic activity and escapes from cell apoptosis, resulting in a pro-tumor and drug-resistant microenvironment for cancer (26). P38, STAT1, and STAT3 are involved in cellular senescence in many cell types (26, 27). Thus overexpression of DUSP26 may inhibit GBM cell senescence and increased cell apoptosis *via* blocking p38 and STAT1. Further studies are needed to verify the mechanism of DUSP26 regulated apoptosis and senescence *via* dephosphorylation of Akt/AMPK and p38/STAT1 signaling pathway. Collectively, our findings suggest that the anti-tumor effects of DUSP26 are manifested through regulation of tumor cell proliferation/migration, apoptosis, and senescence *via* modulating YAP/ERK, Akt/AMPK and/or p38/STAT1 signaling pathways. However, DUSP26 has many other know downstream targets, such as TAK1 (28), FGFR1 (29), and FADD (17), understanding potential role of these targets in the anti-tumor effect of DUSP26 will need further studies. Beyond understanding downstream effects, analyses of upstream regulators of DUSP26 expression have implications for the development of prognostic biomarkers and/or therapeutic approaches. MGMT promoter methylation is the most widely used molecular biomarker that predicts response to standard of care treatment in GBM (30). Similarly, assays to reliably detect DUSP26 or its upstream regulatory events may help delineate the applicability of DUSP26 as a prognostic biomarker for glioma patients.

## Conclusions

**Figure 8:**
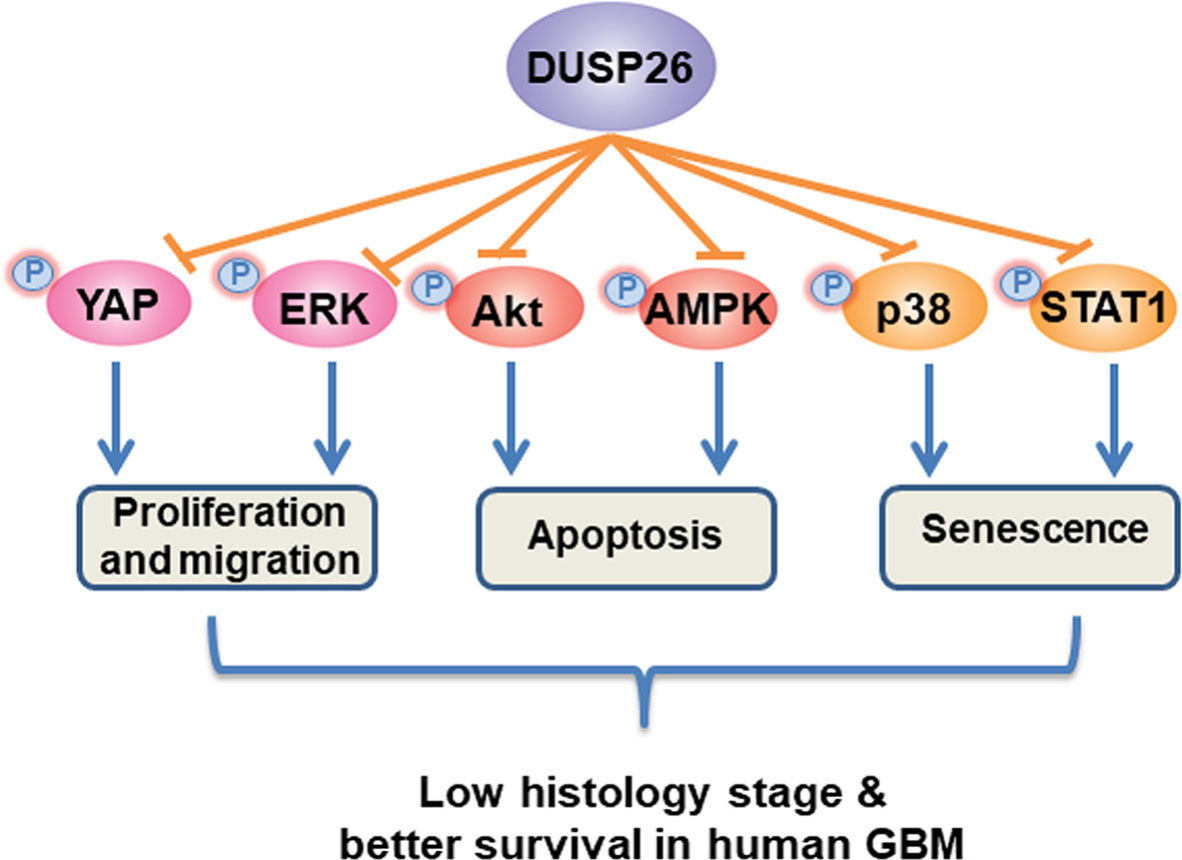
Schematic diagram of proposed mechanism of anti-tumor effects of DUSP26. DUSP26 exerts the anti-tumor effect *via* inhibiting proliferation, migration, and senescence of tumor cells, and by promoting cell apoptosis. DUSP26-YAP/ERK, DUSP26-Akt/AMPK, DUSP26-p38/STAT1 signaling axis are involved in regulating tumor cell proliferation/migration, apoptosis, and senescence, respectively.

As shown in the schematic mechanism model (**Figure 8**), overexpression of DUSP26 suppresses cell proliferation, cell migration, and cell senescence and promotes cell apoptosis. The anti-tumor effect of DUSP26 is associated with inhibition of MAPK and Akt signaling pathways that may arise from dephosphorylation of signaling proteins including, p38, STAT1, ERK, YAP, Akt, and AMPK, which are known substrate of DUSP26 enzymatic activity. With a tight correlation between DUSP26 expression levels and patient survival, DUSP26 can serve as a prognostic marker in glioma patients. Moreover, genetic or pharmacologic tools that increase DUSP26 expression may help design novel therapeutic approaches for GBM.

## Data Availability Statement

The original contributions presented in the study are included in the article/**Supplementary Material**. Further inquiries can be directed to the corresponding author.

## Ethics Statement

The studies involving human participants were reviewed and approved by the ethical committee of Shengjing Hospital, China Medical University. The patients/participants provided their written informed consent to participate in this study.

## Author Contributions

JC conceived and designed the study. JC, HT, RW, YX, and MJ performed research and analyzed the data. JC, HT, and YZ drafted manuscript. All authors contributed to the article and approved the submitted version.

## Funding

This study is supported by the Liaoning Doctoral Science Project (20170520052 and 20180540089), 345 Talent Project of Shengjing hospital, and Natural Science Foundation of China (81802507).

## Conflict of Interest

The authors declare that the research was conducted in the absence of any commercial or financial relationships that could be construed as a potential conflict of interest.
