# Corrigendum: Decreased DUSP26 Expression Promotes Malignant Behavior in Glioblastoma Cells *via* Deregulation of MAPK and Akt Signaling Pathway

**DOI:** 10.3389/fonc.2021.676647

**Published:** 2021-04-12

**Authors:** Jiajia Chen, Yuecan Zeng, Rong Wu, Ying Xuan, Min Jiang, Hao Teng

**Affiliations:** ^1^ Department of Oncology, Shengjing Hospital of China Medical University, Shenyang, China; ^2^ Department of Neurosurgery, Shengjing Hospital of China Medical University, Shenyang, China

**Keywords:** apoptosis, senescence, proliferation, YAP, glioblastoma

In the original article, there was a mistake in **Figure 4**, **Figure 6** and **Figure 7** as published. In **Figure 4**, we put the non-representative picture of wound healing result in U87 DUSP26 plasmid 0h group. In **Figure 6C**, we put the non-representative picture of western result of YAP in U87 group. In **Figure 7B**, we put the non-representative picture of result in U251 DUSP26 plasmid group. These are due to our carelessness in compiling these figures. The corrected **Figure 4**, **Figure 6** and **Figure 7** appear below. In the original article, we neglected to include the funder **Natural Science Foundation of China**, **81802507**.

**Figure 4:**
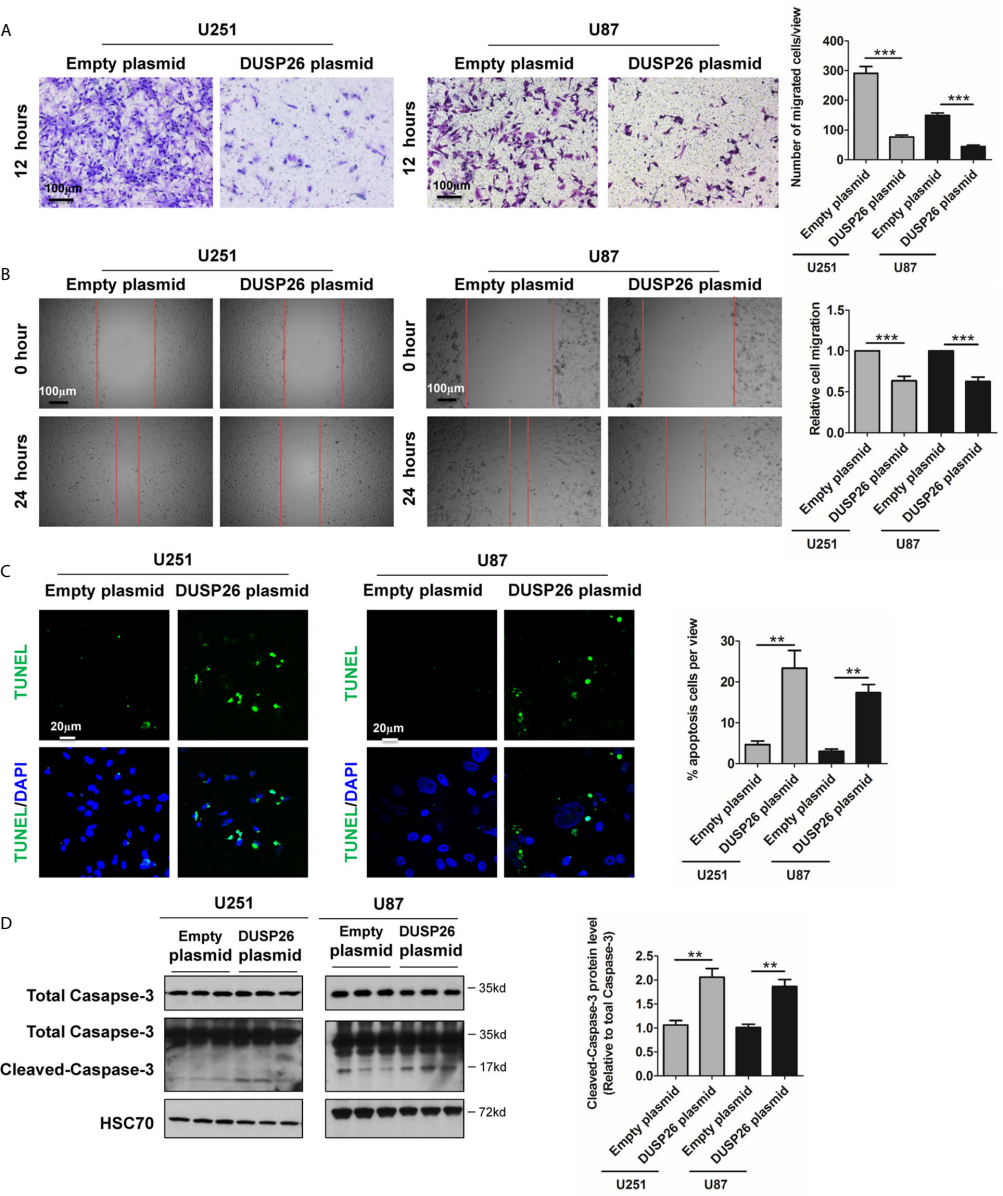
Effect of DUSP26 overexpression on cell migration and apoptosis in glioblastoma (GBM) cells. **(A)** Representative images (left side) and graphs (rightside) showing relative cell migration as determined by transwell migration assays using U251 and U87 cells transfected with an empty plasmid or DUSP26 expression plasmid. Cells were allowed to migrate for 8 h. Cells migrated cells were stained and imaged under a microscope using 10X objective lenses and quantified. **(B)** Photomicrographs showing relative wound healing at 0 and 24 h after the wound was scratched in monolayers of U251 and U87 cells transfected with an empty plasmid or DUSP26 expression plasmid wound closure was measured and quantified. **(C)** Representative images (panels on the left side) and graphs of quantification (panel on the right side) of apoptotic cells detected by TUNEL assay performed using U251 and U87 cells transfected with an empty plasmid or DUSP26 expression plasmid. **(D)** Representative images of western blots (panels on the left side) and showing levels of total caspase-3, cleaved caspase-3 in lysates of U251 or U87 cells transfected with empty vector or DUSP26 plasmid construct, and lysed 48 h after transfection were analyzed in triplicate, HSC70 was used as loading control; graphs depicted on the right side show the quantification of cleaved caspase-3. **p < 0.05; ***p < 0.001.

**Figure 6:**
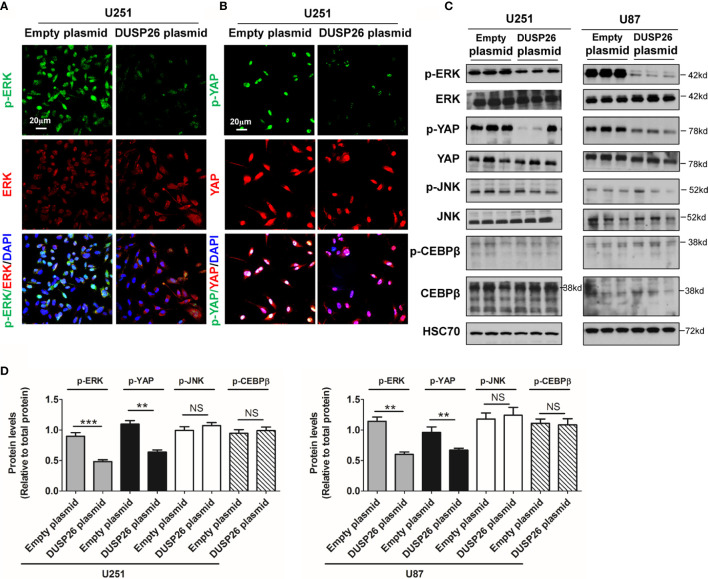
Effect of DUSP26 overexpression on the MAPK signaling pathway. **(A)** Representative images of immunofluorescence based detection of p-ERK (green), ERK (red) in U251 cells transfected with empty vector or DUSP26 expression plasmid, cells were analyzed 48 h after transfection, and nuclei (blue) were counterstained with DAPI. **(B)** Representative images of immunofluorescence based detection of p-YAP (green), YAP (red) in U251 cells transfected with empty vector or DUSP26 expression plasmid and processed as described under **(A)**. **(C)** Representative images of western blots showing levels of p-ERK (Thr202/Tyr204), ERK, p-YAP (Ser127), YAP, p-JNK (Thr183/Tyr185), JNK, p-CEBPb (Thr235), and CEBPb in lysates of U251 or U87 cells transfected with empty vector or DUSP26 plasmid construct, cells were lysed 48 h after transfection and analyzed in triplicate, HSC70 was used as loading control. **(D)** Quantification of the western blots depicted in **(C)**. **p < 0.01; ***p < 0.001 and NS, denotes non-significant.

**Figure 7:**
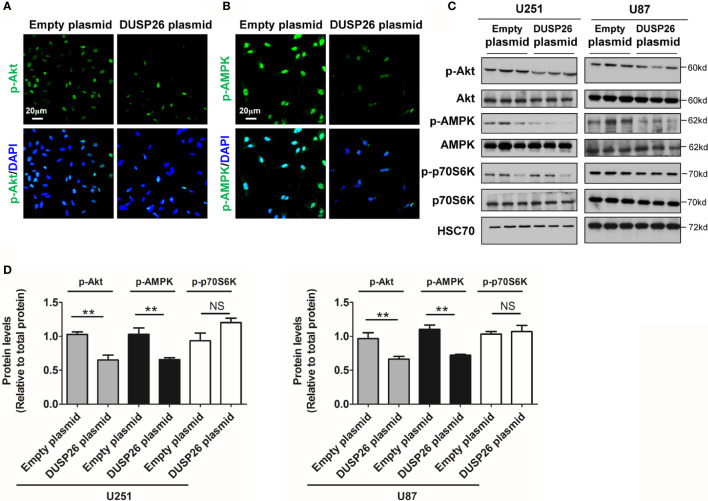
Effect of DUSP26 overexpression on the Akt signaling pathway. **(A)** Representative images of immunofluorescence based detection of p-Akt (green) in U251 cells transfected with empty vector or DUSP26 cassette, cells were analyzed 48 h after transfection, nuclei were counterstained with DAPI. **(B)** Representative images of immunofluorescence based detection of p-AMPK in U251 cells transfected with empty vector or DUSP26 cassette and processed as described under **(A)**. **(C)** Representative images of western blots showing relative p-Akt (Thr308), Akt, p-AMPK (Thr172), AMPK, p-p70S6K (Ser371), and p70S6K in lysates of U251 or U87 cells transfected with empty vector or DUSP26 expression plasmid, cells were lysed 48 h after transfection and analyzed in triplicate, HSC70 was used as a loading control. **(D)** Quantification of the Western blots depicted in **(C)**, **p < 0.01, NS, not significant.

The authors apologize for this error and state that this does not change the scientific conclusions of the article in any way. The original article has been updated.

