# Supplementary material for: Decreased DUSP26 Expression Promotes Malignant Behavior in Glioblastoma Cells via Deregulation of MAPK and Akt Signaling Pathway
Source: Front Oncol. 2021 Feb 25;11:622826. doi: 10.3389/fonc.2021.622826 (PMC7947697; doi:10.3389/fonc.2021.622826)
Supplement: Supplementary file 1 [file Table_1.doc]

**Supplementary table 1 Antibody information**

| Primary antibody | Catalog | Applications |
| --- | --- | --- |
| DUSP26 | Abcam #ab224407 | IF: 1:100;WB:1:1000 |
| Ki67 | Abcam #ab16667 | IF: 1:100 |
| Cyclin D1 | Santa Cruz #sc20044 | WB:1:1000 |
| HSC70 | Santa Cruz #sc7298 | WB:1:2000 |
| Caspase-3 | Cell signaling #9662S | WB:1:500 |
| p38 | Cell signaling #9212S | WB:1:1000 |
| p-p38 (Thr180/Tyr182) | Cell signaling #9211S | IF: 1:100;WB:1:1000 |
| STAT1 | Cell signaling #14994S | IF: 1:100;WB:1:1000 |
| p-STAT1 (Ser727) | Cell signaling #8826S | IF: 1:100;WB:1:1000 |
| STAT3 | Cell signaling #9139S | WB:1:1000 |
| p-STAT3 (Tyr705) | Cell signaling #9145S | WB:1:1000 |
| ERK | Cell signaling #4696S | IF: 1:100;WB:1:1000 |
| p-ERK (Thr202/Tyr204) | Cell signaling #9101S | IF: 1:100;WB:1:1000 |
| YAP | Santa Cruz #sc376830 | IF: 1:100;WB:1:1000 |
| p-YAP (Ser127) | Cell signaling #13008S | IF: 1:100;WB:1:1000 |
| JNK | Cell signaling #9252S | WB:1:1000 |
| p-JNK (Thr183/Tyr185) | Cell signaling #9255S | WB:1:1000 |
| CEBPβ | Cell signaling #3087S | WB:1:1000 |
| p-CEBPβ (Thr235) | Cell signaling #3084S | WB:1:1000 |
| Akt | Cell signaling #9272S | IF: 1:100;WB:1:1000 |
| p-Akt (Thr308) | Cell signaling #4056S | IF: 1:100;WB:1:1000 |
| AMPK | Cell signaling #2532S | IF: 1:100;WB:1:1000 |
| p-AMPK (Thr172) | Cell signaling #2531S | IF: 1:100;WB:1:1000 |
| p70S6K | Cell signaling #9202S | WB:1:1000 |
| p-p70S6K (Ser371) | Cell signaling #9208S | WB:1:1000 |

**Supplementary table 2 Relationships between DUSP26 expression and cell markers in 180 GBM** cases

| **Molecules** | **DUSP26 expression (%)** | | ***p* value** |
| --- | --- | --- | --- |
|  | **Low** | **High** |  |
| Ki67 |  |  |  |
| <5% | 57 (63.3) | 69 (76.7) | 0.037 |
| ≥5% | 33 (36.7) | 21 (23.3) |  |
| EGFR |  |  |  |
| <5% | 33 (36.7) | 26 (28.9) | 0.170 |
| ≥5% | 57 (63.3) | 64 (71.1) |  |
| PDL1 |  |  |  |
| <5% | 78 (86.7) | 72 (80.0) | 0.159 |
| ≥5% | 12 (13.3) | 18 (20.0) |  |
| GFAP |  |  |  |
| - - + | 39 (43.3) | 45 (50.0) | 0.228 |
| ++ - +++ | 51 (56.7) | 45 (50.0) |  |
| S100 |  |  |  |
| - - + | 53 (58.9) | 50 (55.6) | 0.382 |
| ++-+++ | 37 (41.1) | 40 (44.4) |  |
| CD34 |  |  |  |
| - - -/+ | 79 (87.8) | 83 (92.2) | 0.228 |
| + - ++ | 11 (12.2) | 7 (7.8) |  |
| EMA |  |  |  |
| - | 37 (82.2) | 28 (84.8) | 0.504 |
| + | 8 (17.8) | 5 (15.2) |  |
| CK |  |  |  |
| - - + | 35 (89.7) | 23 (71.9) | 0.052 |
| ++-+++ | 4 (10.3) | 9 (28.1) |  |
| Vimentin |  |  |  |
| - - + | 17 (47.2) | 10 (52.6) | 0.461 |
| ++-+++ | 19 (52.8) | 9 (47.7) |  |
